# A protocol of systematic review and meta-analysis of continuous vacuum sealing drainage for diabetic foot ulcer

**DOI:** 10.1097/MD.0000000000020541

**Published:** 2020-06-19

**Authors:** Xiao-gang Bai, Jing Wang, Xia Li, Wei Li, Jie Xu

**Affiliations:** Department of Endocrine and Metabolism, Yanan University Affiliated Hospital, Yan’an, 716000, China.

**Keywords:** continuous vacuum sealing drainage, diabetic foot ulcer, effectiveness, randomized controlled trial, safety

## Abstract

**Background:**

: Although continuous vacuum sealing drainage (CVSD) is often reported for the management of diabetic foot ulcer (DFU), its effectiveness has been evaluated primarily via clinical outcomes with limited attention. This study will aim to assess the effectiveness of CVSD for the treatment of patients with DFU.

**Methods:**

We will carry out a search in Cochrane Library, MEDLINE, Embase, CINAHL, PsycINFO, Web of Science, Allied and Complementary Medicine Database, Chinese Biomedical Literature Database, and China National Knowledge Infrastructure from their inceptions to the March 1, 2020. We will exert the searches in those electronic databases without restrictions of language, and the use of validated filters to obtain eligible studies. In addition to the search in electronic databases, we will retrieve studies from conference proceedings, and reference lists of included trials. We will use Cochrane risk of bias tool to assess study quality for each eligible study. All statistical analysis will be conducted using RevMan 5.3 software.

**Results:**

This study will systematically summarize the most present evidence to assess the effectiveness and safety of CVSD for the management of DFU.

**Conclusion:**

The results from this study will contribute to obtain a genuine understanding of perspective from evidence-based medicine and a scientific basis for the effectiveness and safety of CVSD for the treatment of DFU.

**PROSPERO registration number:**

PROSPERO CRD42020170723.

## 1 Introduction

Diabetes mellitus is one of the most prevalent diseases worldwide.^[1,2]^ It has many complications; diabetic foot ulcer (DFU) is one of the prominent problems of them.^[3,4]^ It is reported that DFU accounts for 21% to 30% of all 425 million people globally.^[5–8]^ Of those, more than 50% patients with DFU become infected, and 20% of infections lead to amputation.^[9,10]^

Data mining in the medical records revealed continuous vacuum sealing drainage (CVSD) has become a potential management for DFU.^[11–22]^ However, to our best knowledge, no systematic review on the CVSD for DFU has been reported. Herein, a critical appraisal of the available evidence is need, and this proposed study will assess the efficacy and safety of CVSD for treating patients with DFU.

## 2 Methods and analysis

### 2.1 PROSPERO registration

We been registered this study on PROSPERO (CRD42020170723). We will report this study based on the Preferred Reporting Items for Systematic Reviews and Meta-Analysis (PRISRMA) Protocol statement.^[23]^

### 2.2 Study inclusion and exclusion criteria

#### 2.2.1 Types of studies

We will only include randomized controlled trials (RCTs) that focusing on assessing the effectiveness and safety of CVSD for the management of DFU. We will not apply any limitations to language and time of publication.

#### 2.2.2 Types of interventions

Experimental group: We will include all patients who utilized CVSD as their treatment.

Control group: We will consider patients who received other treatments as their management, except CVSD.

#### 2.2.3 Types of participants

All patients who were diagnosed as DFU will be included. We will not imply their race, gender, age, and economic status.

#### 2.2.4 Types of outcome measurements

The primary outcomes are change in ulcer area (surface area change in cm^2^ since baseline), and time to complete healing (complete healing was defined as intact skin).

The secondary outcomes consist of proportion of ulcers healed within trial period, quality of life (as measured by any validated scale), pain intensity (as measured using any validated tools), and adverse events (as reported by the trial author, such as infection)

### 2.3 Search methods for the identification of studies

In this study, we will systematically conduct a comprehensive search from the electronic databases of Cochrane Library, MEDLINE, Embase, CINAHL, PsycINFO, Web of Science, Allied and Complementary Medicine Database, Chinese Biomedical Literature Database, and China National Knowledge Infrastructure from their inceptions to the March 1, 2020. No limitations of language and time of publication will be imposed to any electronic databases search. The sample of search strategy for Cochrane Library has been created and is shown in Table 1. We will adapt similar search strategies for other electronic databases. Besides the sources of above electronic databases, we will perform search from the conference proceedings, and reference lists of included studies.

**Table 1:**
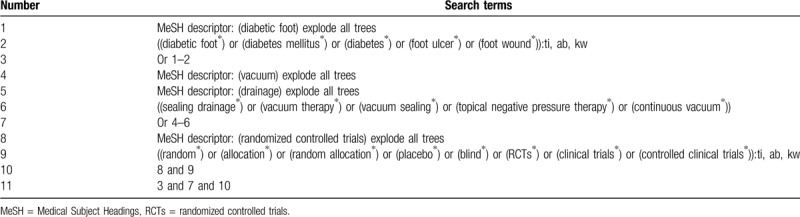
Search strategy for Cochrane Library database.

### 2.4 Data collection and analysis

#### 2.4.1 Selection of studies

Reference manager software will be used to manage the search results, and all irrelevant studies will be excluded. Then, the titles and abstracts of all literatures will be screened against the inclusion criteria by 2 independently researchers. When necessary, we will plan to obtain a copy of potential eligible trials to determine their final inclusion. We will report selection process using PRISMA flowchart and will note the reasons for all ineligible studies in a specific table. Any divergences between 2 researchers will be resolved by a 3rd researcher through discussion.

#### 2.4.2 Data extraction and management

Before the data extraction, we will build a standardized data collection sheet and then will verify the structure of form and the suitability of the process using at least 3 studies. All process of data extraction will be performed by 2 independent researchers. If any different opinions between 2 researchers occur, we will consult a 3rd researcher to make final decision. All following information will be collected: study information (including title, 1st author, publication year, country, etc), patient information (including age, sex, sample size, diagnostic criteria, eligibility criteria, etc), study methods (descriptions of randomization, blind, etc), managements in both experimental and control groups (intervention types, dosage, mode, delivery method, etc), outcomes, safety, and any other essential information.

#### 2.4.3 Missing data dealing with

If we identify any missing or unclear information, we will contact primary authors to obtain them. If not, we will only analyze available data, and will discuss its potential impacts as a limitation.

### 2.5 Study quality assessment

Two independent researchers will investigate study quality for all included trials using Cochrane risk of bias tool. It covers 7 different areas, and each domain is judged as low risk of bias, unclear risk of bias, or high risk of bias. A 3rd researcher will help to solve any different views between 2 researchers.

### 2.6 Statistical analysis

All statistical analysis will be performed using RevMan 5.3 software. As for continuous data, we will use mean difference or standard mean difference and 95% confidence intervals to calculate it. As for dichotomous data, we will utilize risk ratio and 95% confidence intervals to present it. We will use *I*^2^ statistic to explore potential heterogeneity among included trials. It will be explained as follows: *I*^2^ ≤ 50% indicating reasonable heterogeneity, while *I*^2^ > 50% presenting obvious heterogeneity. We will apply a fixed-effects model to pool the data if *I*^2^ ≤ 50%, and a meta-analysis will be undertaken if it is possible. On the contrary, we will use a random-effects model to synthesize the data if *I*^2^ > 50%, and subgroup analysis will be carried out to identify any possible reasons for such obvious heterogeneity. If necessary, we will also undertake a narrative summary to address and report the merged outcome data.

### 2.7 Additional analysis

#### 2.7.1 Subgroup analysis

Subgroup analysis will be conducted in accordance with the characteristics of study or patient, study methods, managements, and outcomes.

#### 2.7.2 Sensitivity analysis

Sensitivity analysis will be performed to identify the robustness of merged results by excluding low quality studies.

#### 2.7.3 Reporting bias

If at least 10 trials are included, we will undertake funnel plot and Egger regression test to check if there are any reporting biases.^[24,25]^

### 2.8 Ethics and dissemination

This study will not need any ethical documents, because we will not utilize any individual patient data. The findings of this study are expected to be published on a peer-reviewed journal or presented in a conference meeting.

## 3 Discussion

Previous studies have reported that the effectiveness and safety of CVSD for the management of DFU. However, all evidence is still at the conceptual level, and no systematic review has been performed regarding the effectiveness and safety of CVSD for DFU. In this study, all potential eligible studies regarding the CVSD for the treatment of DFU will be fully synthesized without language and publication time limitations. The findings of this study may yield helpful evidence for clinical practice and future studies.

## Author contributions

**Conceptualization:** Jing Wang, Xia Li, Jie Xu.

**Data curation:** Jing Wang, Xia Li, Wei Li.

**Formal analysis:** Xiao-gang Bai, Jing Wang, Wei Li, Jie Xu.

**Investigation:** Xia Li.

**Methodology:** Xiao-gang Bai, Jing Wang, Wei Li, Jie Xu.

**Project administration:** Xia Li.

**Resources:** Xiao-gang Bai, Jing Wang, Wei Li, Jie Xu.

**Software:** Xiao-gang Bai, Jing Wang, Wei Li, Jie Xu.

**Supervision:** Xia Li.

**Validation:** Xiao-gang Bai, Jing Wang, Xia Li.

**Visualization:** Xiao-gang Bai, Xia Li, Wei Li, Jie Xu.

**Writing – original draft:** Xiao-gang Bai, Xia Li, Wei Li, Jie Xu.

**Writing – review & editing:** Xiao-gang Bai, Jing Wang, Xia Li, Jie Xu.
